# Novel phytoandrogens and lipidic augmenters from *Eucommia ulmoides*

**DOI:** 10.1186/1472-6882-7-3

**Published:** 2007-01-29

**Authors:** Victor YC Ong, Benny KH Tan

**Affiliations:** 1 Department of Pharmacology, Yong Loo Lin School of Medicine, National University of Singapore, 10 Kent Ridge Crescent, 119260, Singapore; 2 Faculty of Medicine, Nursing and Health Sciences, Monash University, Wellington Road, Victoria 3800, Australia

## Abstract

**Background:**

Plants containing compounds such as the isoflavonoids, with female hormone-like effects that bind to human estrogen receptors, are known. But none has been previously shown to have corresponding male hormone-like effects that interact with the human androgen receptor. Here, we report that the tree bark (cortex) of the Gutta-Percha tree *Eucommia ulmoides *possesses bimodal phytoandrogenic and hormone potentiating effects by lipidic components.

**Methods:**

The extracts of *E. ulmoides *were tested using *in-vitro *reporter gene bioassays and *in-vivo *animal studies. Key compounds responsible for the steroidogenic effects were isolated and identified using solid phase extraction (SPE), high performance liquid chromatography (HPLC), thin layer chromatography (TLC), gas chromatography-mass spectroscopy (GC-MS), electron spray ionisation-mass spectroscopy (ESI-MS) and nuclear magnetic resonance (NMR).

**Results:**

The following bioactivities of *E. ulmoides *were found: (1) a phenomenal tripartite synergism exists between the sex steroid receptors (androgen and estrogen receptors), their cognate steroidal ligands and lipidic augmenters isolated from *E. ulmoides*, (2) phytoandrogenic activity of *E. ulmoides *was mediated by plant triterpenoids binding cognately to the androgen receptor (AR) ligand binding domain.

**Conclusion:**

In addition to well-known phytoestrogens, the existence of phytoandrogens is reported in this study. Furthermore, a form of tripartite synergism between sex steroid receptors, sex hormones and plant-derived lipids is described for the first time. This could have contrasting clinical applications for hypogonadal- and hyperlipidaemic-related disorders.

## Background

The androgen receptor (AR) plays a pivotal role in human (both male and female) physiology such as skeletal muscle development, bone density, fertility and sex drive [1,2]. The α and β estrogen receptors (ERs), likewise, have fundamental impact on the sex hormone-mediated physiological milieu. Conversely, over-active sex steroid (androgen and estrogen) receptors have been linked to increased risks of hormone-sensitive tumours such as prostate and breast cancers. Availability and binding of cognate ligands to the ligand binding domain (LBD) of the sex steroid receptors are required for the proportionate expression of specific genes responsible for such sex hormone-mediated processes [3,4].

Vegetative foods such as the legumes, particularly soybean (*Glycine max*), contain phytoestrogens that modulate the transcriptional activities of the estrogen receptor isoforms, α and β. The former has been linked to the chemoprevention of specific cancers in the breast and prostate gland [5].

**Figure 1:**
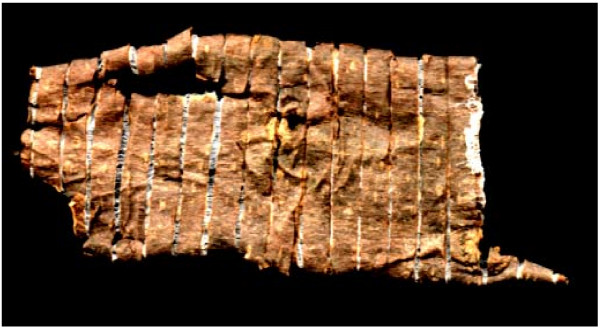
Processed bark (cortex) from the evergreen tree *Eucommia ulmoides*. Originating from temperate regions of China, botanical parts of *E. ulmoides *such as the leaves and bark are used medicinally in the Chinese and Japanese Pharmacopia. Note the silvery threads of resin (Gutta Percha) in between the sliced portion of the specimen (green arrow).

Here, we report that the tree bark (cortex) of the Gutta-Percha tree *Eucommia ulmoides *OLIVER possesses novel bimodal phytoandrogenic and synergistic augmentation of hormone-dependent receptor activity. *E. ulmoides *is also known variously as the Gutta Percha Tree, the Rubber Bark Tree or Du-Zhong [6,7]. The toothed elliptic leaves and tree bark of *E. ulmoides *(figure 1) are used medicinally in herbal pharmacopoeias such as Kampo (traditional Japanese medicine) and Zhong-Yao (traditional Chinese medicine) for indications such as the relief of back pain, to increase stamina, to make bones and muscles 'strong' and to hasten recovery from fatigue. It is noted that these are male hormone-related pharmacological effects.

Through the combined use of varied technologies – recombinant DNA constructs, reporter gene assays, animal studies and separation chemistry, extracts of *E. ulmoides *were shown for the first time, to specifically activate the tranactivational capacity of the sex steroid receptors in both *in-vitro *and *in-vivo *settings. A series of bioassay-guided fractionation showed that the phytoandrogenic and hormone potentiating effects of *E. ulmoides *were mediated by distinct groups of phytocompounds; triterpenoids and short-chain lipids respectively.

## Methods

### Cell culture

The mammalian COS-7 cell line was maintained in DMEM cell culture medium with 10% fetal bovine serum (FBS) and 1% of each amino acids (glutamine, arginine and lysine). Hela cells were maintained in RPMI 1640 cell culture medium (with 10% FBS, 1% L-glutamine, 1% L-lysine and 1% arginine). Both cell lines were incubated at 37°C in 5% CO_2 _gaseous environment.

### Transient transfection of mammalian cell lines

Mutant and wild-type (WT) chimeric constructs were transfected into Hela cells, using the lipofection technique. Both are as described in [8]. Hela cells, which are a homologous cell line, was utilised for the transactivation studies. These Hela cells were cotransfected with a luciferase reporter gene containing two AREs [p(ARE)_2_-Tata-Luc]. Transfected cells were exposed to androgens for 48 hr before harvesting and quantification of luciferase activity. The androgens used were testosterone and dihydrotestosterone (DHT).

### Binding site of phytoandrogens

Radioligand displacement assays were carried out using tritiated testosterone to determine binding site of the phytoandrogens in *E. ulmoides*. The COS-7 cells were transfected with AR and then exposed to 3 nM of tritiated testosterone and the indicated amounts of DHT (nM), cortisol (nM) or ethanolic *E. ulmoides *(EU) extract (1 concentration factor (c.f.) = 50 ng dry weight/ml treatment medium) for 2 hr at 37°C. The treated cells were harvested and the amount of tritiated testosterone bound to AR was then measured by scintillation counting. Specific binding is expressed as percent tritium bound to AR, where 100% is the amount of specific tritiated-testosterone bound in the absence of competing cold ligand minus background (non-specific binding to substrate and proteins). Each data point, the mean of quadruplicates, represents the amount of radiolabelled testosterone specifically bound on exposure to indicated doses of DHT, ethanolic EU extract or cortisol.

### Luciferase assay

Luciferase assay was performed with the TD-20/20 Luminometer, following the instructions provided by the manufacturer (Promega, USA). Briefly, the transfected cells were lysed in 1× Reporter Lysis Buffer (RLB). The cell lysate was mixed with Luciferase Assay Substrate immediately before measuring. The luciferase activity was measured in relative light units (RLU).

### Plant material

The dried cortex of *Eucommia ulmoides *OLIVER was purchased from the local wholesaler dealing exclusively in Chinese medicinal herbs. Dr Ruth Kiew, Keeper of Herbarium & Library, National Parks Board, Singapore, authenticated the plant material. A voucher specimen (BT-4) was deposited with the Singapore Botanic Gardens Herbarium.

### Extractions

Dried cortical barks of *E. ulmoides *were washed with deionised water to remove contaminants. In each batch extraction, damp *E. ulmoides *barks were then macerated using a commercial grinder (Bosch, Germany). The resulting cortical mash was then blended with 100% ethanol solvent. Using the soak method, the blended ethanolic mash (100 g/1L) was incubated in a shaken (25 r.p.m.) water bath (Buchi Labortechnik AG, Switzerland) for 12 hr at 40°C to allow the active compounds to dissolve into the solvent system. After 12 hr, the menstrum was decanted and filtered through Whatman No.1 using a Buchner funnel *in vacuo*. The resulting filtrate was concentrated by evaporation under vacuum at 40°C, using a rotatory evaporator (Buchi Labortechnik AG, Switzerland). The extraction procedure was repeated 8 to 10 times until the fibrous residue was exhausted. The menstrums collected from each sequential re-extraction were concentrated and blended into a semi-solid paste. This paste was then aliquoted and resuspended in pure ethanol to obtain stock solutions (50 mg/ml) of tertiary crude extracts for downstream experimental work.

### Animal housing

Male juvenile (prepubertal) WISTAR rats weighing between 48–64 g (average weight 53 g) were obtained from the Laboratory Animal Centre, National University of Singapore. The animals were fed on commercial pelleted chow and water was given *ad libitum*. The rats were housed individually in wide-bottomed Perspex cages. The light cycle was diurnal with 12 hr daylight. The animals were treated in accordance with the "guidelines for the care and use of laboratory animals for scientific purposes" issued by the local authority.

### Animal studies

To establish the dose response relationship between intramuscular (IM) testosterone (Sustanon 5000) injections and ventral prostate weight (as a measure of prostate development), prepubertal male WISTAR rats (2 weeks old) were given IM testosterone injections of 500 μg, 2500 μg and 5000 μg while control animals were given IM injections of vehicle (olive oil) only. After 5 days, the WISTAR rats were sacrificed by decapitation. The abdominal flap of each rat was dissected to recover the prostate gland. The ventral prostate gland was removed and the wet weight determined. Androgen-mediated prostatic growth was expressed as the weight of the ventral prostate gland normalized to 100 g body weight of the individual rats.

To study the androgenic effect of oral *E. ulmoides *liquid formulation on ventral prostate growth, prepubertal male WISTAR rats were administered *E. ulmoides *extracts via gavaging with doses of 1 mg, 5 mg, 10 mg and 50 mg in aliquots of 1 ml vehicle (25% hydroethanolic solution). Gavaging (gastric intubation) were carried out with gauge 18 feeding needle. Negative control animals were given oral doses of vehicle (25% hydroethanolic solution) only.

To test the potentiating effect of *E. ulmoides *extract on testosterone-mediated prostatic growth, prepubertal male WISTAR rats were given 5000 μg IM testosterone injections in conjunction with 50 mg of *E. ulmoides *extract by oral gavage. Baseline control animals were given IM injections of 5000 μg IM testosterone injections alone. Negative control animals were given IM injections of olive oil plus concurrent oral gavaging with 25% hydroethanolic solution.

### Fractionations

Semi-preparative open column chromatography was carried out using diol stationary phase as previously described [10]. The chromatographic column with the *E. ulmoides *extract loaded on top was influxed with 50 ml of each mobile phase in increasing order of polarity – dichloromethane (DCM), ethyl acetate (EA), ethanol (EtOH) and water (H_2_O). The mobile phases eluting from the chromatographic column were collected sequentially into individual fractions – fraction A, fraction B, fraction C and fraction D. These fractions were dried in a rotary evaporator at 40°C and resuspended in ethanol for bioassays.

For solid phase extraction (SPE), the SPE C-18 cartridge was flushed with ethanol to activate the C-18 hydrocarbon moieties. This was followed by water to displace the ethanol phase and equilibrate the solid phase in an aqueous environment. Using SPE method described in Ong [10], fraction A was further resolved into four purer fractions AA, AB, AC and AD. Fraction AB with dominant phytoandrogenic activity was subsequently re-chromatographed in C-18 matrix using high performance liquid chromatography (HPLC) to obtain a phytoandrogenic fraction (AB-P1) of even greater purity. AB-P1 fraction was subjected to thin layer chromatography (TLC) and a phytoandrogenic triterpenoid fraction TLC4-5 was recovered for molecular mass measurement using electron spray ionization-mass spectroscopy (ESI-MS). The TLC chromatogram of fraction AB-P1 was subjected to vanillin-sulphuric acid visualization reaction to detect presence of triterpenoids by spraying 5% ethanolic sulphuric acid. This was followed by 1% ethanolic vanillin. Colorimetric development was completed by heating the treated TLC plate at 120°C for 15 min in a thermostat oven.

**Figure 2:**
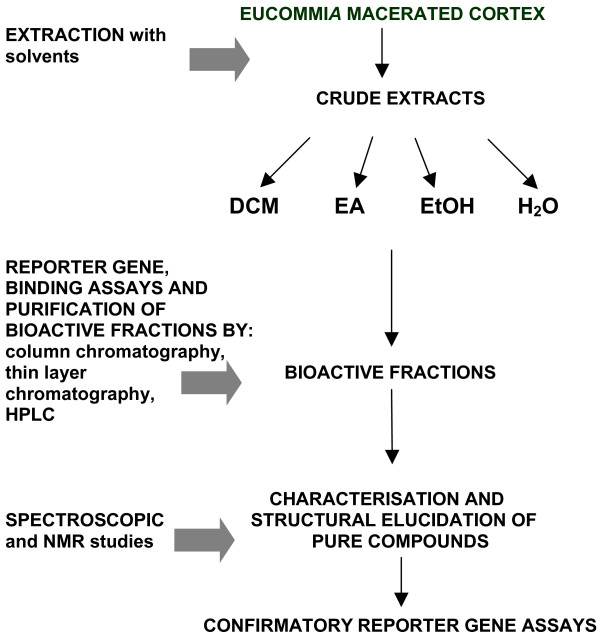
Schematics of research process flow. *E. ulmoides *cortex was macerated and crude extracts obtained using solvents. These solvents were dichloromethane (DCM), ethyl acetate (EA), ethanol (EtOH) and water (H_2_O). The crude extracts were subjected to steroidogenic reporter gene and binding assays. Bioactive fractions were subsequently obtained through bioassay-guided fractionations using column chromatography, thin layer chromatography and high performance liquid chromatography (HPLC). Pharmacologically active phytocompounds purified from the bioactive fractions were identified through spectroscopic and nuclear magnetic resonance (NMR) studies. Confirmatory reporter gene assays were then carried out using pure compounds to reproduce steroidogenic effects.

Fraction C was similarly subjected to SPE using C-18 SPE cartridge and sequential fractions recovered were fractions CA and CB. The recovered fraction CB was subjected to ^1^H NMR and GC-MS [10]. The overall scheme, from raw herb to confirmatory bioassays, is detailed in figure 2.

### Statistical analyses

All *in-vitro *reporter gene results were expressed as means ± SE from triplicate assays. Results of the radioligand displacement assays were expressed as means ± SE from quadruplicate assays. One-way ANOVA analyses were carried out to assess statistical significance for *in-vivo *animal experiments. Differences with p-value < 0.001 were considered statistically significant.

## Results and discussion

**Figure 3:**
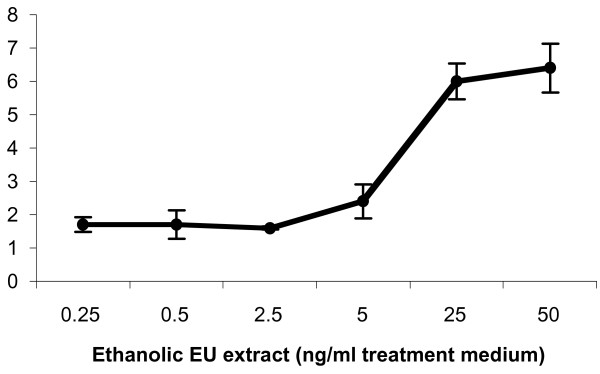
Phytoandrogenicity of *E. ulmoides *(EU) extract. The dose response curve of an *E. ulmoides *extract with concentrations ranging from 0.25 ng/ml treatment medium to 50 ng/ml treatment medium. Hela cells transiently expressing androgen receptor (AR) in the presence of AR-responsive luciferase reporter gene (Luc) were exposed to increasing doses of the extract. Comparatively, testosterone (1 nM) has 100-fold Luc activity relative to the maximal *E. ulmoides *6.4-fold Luc activity. Data are mean ± SE of three replicates.

**Figure 4:**
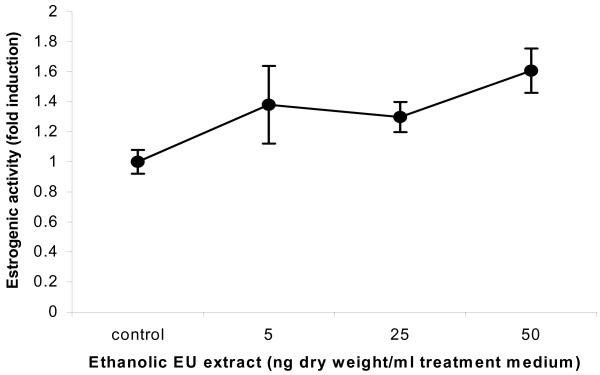
Phytoestrogenicity of *E ulmoides *(EU) extract. Phytoestrogenic effect of *E ulmoides *(EU). Hela cells were transfected with human estrogen receptor and estrogenic effect of an ethanolic EU extract measured with MMTV-ERE-Luc reporter gene. Data are mean ± SE of three replicates.

**Figure 5:**
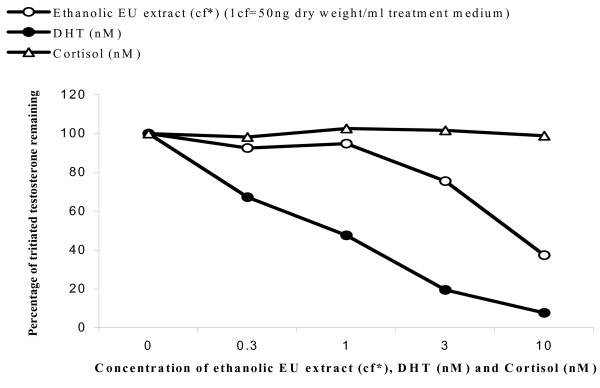
Radioligand displacement assay of *E. ulmoides *(EU) extract. Tritiated testosterone was incubated with transfected Hela cells transiently expressing AR protein. Concentration-dependent competitive displacement of tritiated testosterone by androgenic *E. ulmoides *(EU) extract (1c.f. = 50 ng/ml treatment medium) was measured by the scintillation count of the bound radioisotopic testosterone to AR protein. DHT (nM) and cortisol (nM) acted as positive and negative controls respectively. Data are mean ± SE of four replicates.

**Figure 6:**
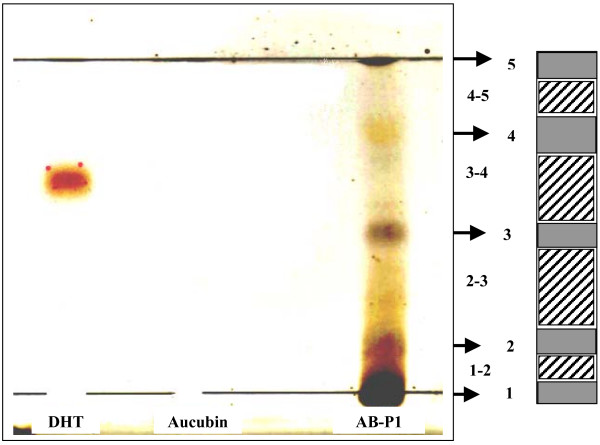
TLC silica fractionation of fraction AB-P1 and triterpenoid detection. Lane 1 (DHT) = 20 μl of 1 mM dihydrotestosterone spotted on, band-wise. Lane 2 (Aucubin): 20 μl of 10 uM aucubin spotted on, band-wise. Lane 3 (AB-P1): 20 μl of fraction AB-P1 (1c.f.) spotted on, band-wise. Colorimetric development was carried out using vanillin-sulphuric acid reaction to detect presence of triterpenoids.

**Figure 7:**
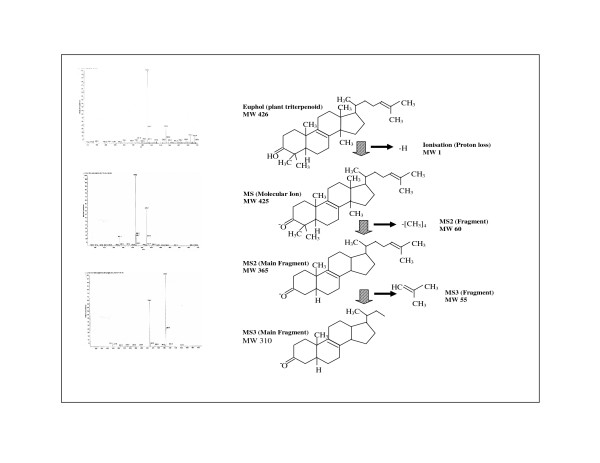
Modeled fragmentation pattern of phytoandrogen structure using Euphol (MW 426), a plant triterpenoid. The ESI-MS spectra of phytoandrogen fraction TLC4-5 indicated a MW of 426. Molecular ions – 1) MS1 = 425, MS2 = 365, MS3 = 310. Euphol (MW 426) was used to model the ESI-MS spectra of the phytoandrogen TLC4-5.

In Luc reporter gene bioassays, *E. ulmoides *cortical extract demonstrated androgenic and estrogenic activities by weakly activating AR and ER transactivational function in a dose-dependent manner (figures 3 and 4). Highly specific radioisotopic ligand displacement bioassay showed that phytocompounds in the *E. ulmoides *extract were able to compete with and displace bound ^3^H-labelled testosterone from the AR ligand binding domain (figure 5). Subsequent bioassay-guided purification of the androgenic extract using chromatographic and ESI-MS techniques revealed that this phytoandrogenic activity was being mediated by triterpenoids, which differs from the phytoestrogenic effect exerted by isoflavonoids [5] (figures 6 and 7).

**Figure 8:**
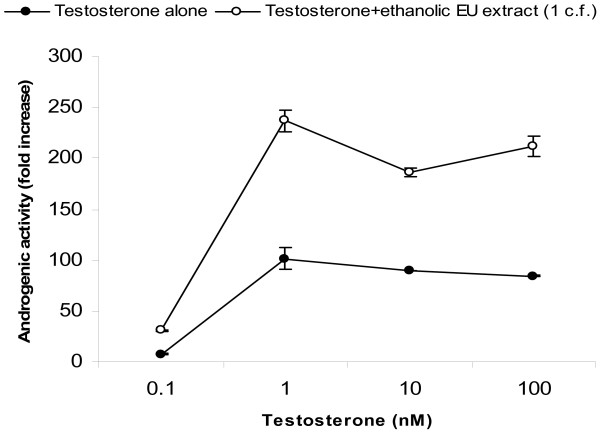
Supra-hormonal effect of *E. ulmoides *(EU) extract on androgen receptor and testosterone. Testosterone-augmenting activity of a fixed dose of ethanolic EU extract (1c.f. = 50 ng/ml treatment medium) with increasing doses of testosterone. Hela cells transiently expressing androgen receptor (AR) in the presence of AR-responsive luciferase reporter gene (Luc) were exposed to increasing doses of EU extract. Data are mean ± SE of three replicates.

**Figure 9:**
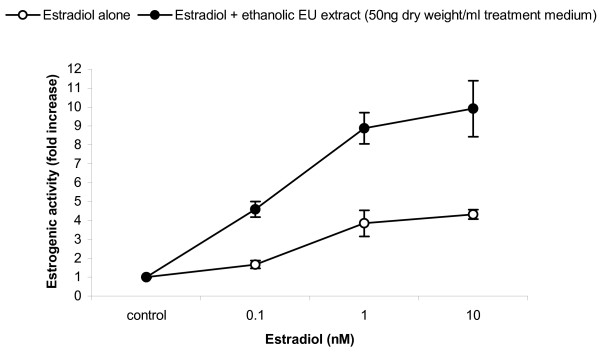
Supra-hormonal properties of *E ulmoides *(EU) extract on estrogen receptor and estradiol. The augmenting effect of a fixed dose of *E ulmoides *(EU) extract on ER. Cells were exposed to increasing doses of estradiol, with or without, 50 ng/mL treatment medium of the ethanolic *E ulmoides *extract, as indicated. Control cells were not exposed to the ethanolic *E ulmoides *extract or estradiol. Estrogenic activity is expressed as fold increase in reporter gene activity compared to control. Data are mean ± SE of three replicates.

Remarkably, combination of DHT and the *E. ulmoides *extract in the presence of the AR led to increases in AR-mediated reporter gene expression ranging from 112% to 204%, even at saturating levels of DHT (figure 8). This potentiating effect was a tripartite synergism between the AR protein, its cognate steroidal ligand and *E. ulmoides*'s extract. This is highly unusual as normally, androgen-mediated AR transcriptional capacity, akin to all ligand-dependent steroid receptors, plateaus at saturating doses of its cognate ligand. A similar synergistic effect was observed when *E. ulmoides *extract was tested in combination with estradiol in the presence of the estrogen receptor (ER) α (figure 9).

To confirm the specificity of these agonistic and synergistic interactions between the sex steroid receptors and *E. ulmoides *cortical extract, the progesterone (PR) and glucocorticoid (GR) receptors (which belong to the same superfamily of steroid receptors) were also subjected to the same series of reporter gene bioassays (data not shown). The cortical extract did not exert any activating effects, alone or in combination with progesterone and cortisol on the PR and GR respectively. This validates the specificity of *E. ulmoides*'s sex steroidogenic effects on the holo-androgen and estrogen receptors.

**Figure 10:**
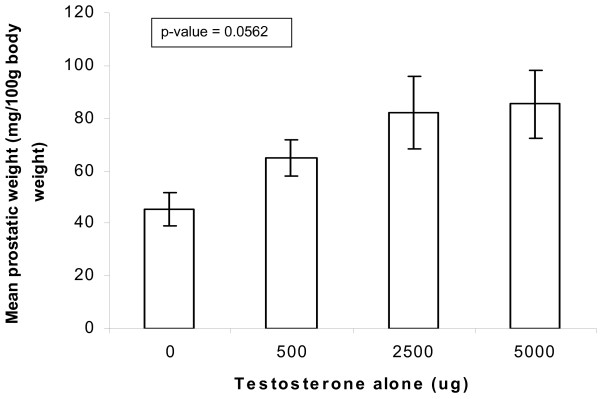
*In-vivo *testosterone and ventral prostate growth in prepubertal male WISTAR rats. The establishment of a dose response relationship between IM testosterone injections and ventral prostatic weight (as a measure of prostate development). Prepubertal male WISTAR rats were given IM testosterone injections of 500 μg, 2500 μg and 5000 μg. Control animals were given IM injections of vehicle (olive oil) only. Prostatic growth is expressed as the weight of the ventral prostate gland normalized to 100 g body weight of the individual rats. Each data point is mean ± SE of five animals' ventral prostate weights.

**Figure 11:**
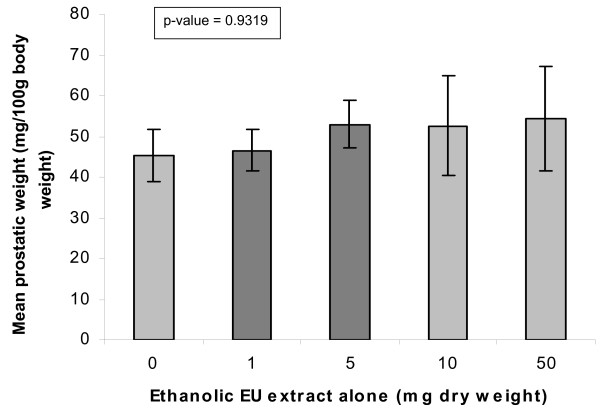
*In-vivo E. ulmoides *(EU) extract and ventral prostate growth in prepubertal male WISTAR rats. The androgenic effect of oral *E. ulmoides *liquid formulation on ventral prostate growth. Prepubertal male WISTAR rats were administered ethanolic *E. ulmoides *(EU) extracts via gavaging with doses of 1 mg, 5 mg, 10 mg and 50 mg. Control animals were given oral doses of vehicle (ethanolic water) only. Prostatic growth is expressed as the weight of the ventral prostate gland normalized to 100 g body weight of the individual rats. Each data point is mean ± SE of five animals' ventral prostate weights.

*In-vivo *animal studies were then carried out to investigate the androgenic and hormone potentiating effects of *E. ulmoides *extract when it is administered orally. At baseline (without testosterone), the mean ventral prostate weight of the male Wistar rats was 45 mg/100 g body weight. With a saturating dose of 5000 μg IM testosterone injection, the androgen-mediated ventral prostate growth steadied at 85 mg prostate weight/100 g body weight; one-way ANOVA showing statistically insignificant difference (p-value = 0.0562) (figure 10). Administration of *E. ulmoides *oral formulations at dry weight doses of 1 mg, 5 mg, 10 mg and 50 mg (n = 5 for each dosage) similarly brought about a statistically insignificant increase over baseline (p-value = 0.9319) at the maximum dose of 50 mg (figure 11).

**Figure 12:**
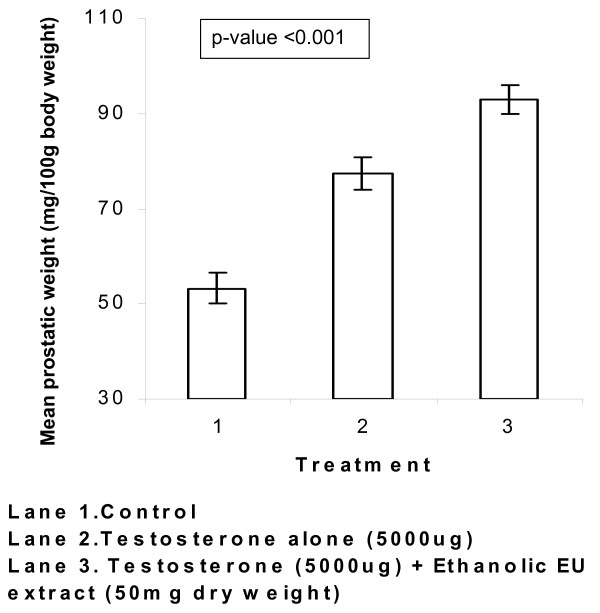
*In-vivo *testosterone-*E. ulmoides *(EU) extract and ventral prostate growth in prepubertal male WISTAR rats. The synergistic augmenting effect of *E. ulmoides *on testosterone mediated prostate growth. Prepubertal male WISTAR rats were given IM testosterone injections of 5000 μg (Lane 2). To test synergism between *E. ulmoides *and an androgen, experimental rats were given saturating IM testosterone injections of 5000 μg in conjunction with oral gavaging of 50 mg of *E. ulmoides *(EU) extract (Lane 3). Control animals were given IM injections of olive oil plus concurrent oral gavaging of ethanolic water. Prostatic growth is expressed as the weight of the ventral prostate gland normalized to 100 g body weight of the individual rats. Each data point is mean ± SE of five animals' ventral prostate weights.

However, a combined administration of 5000 μg IM testosterone injection and *E. ulmoides *by oral gavaging (N = 15) augmented androgen-mediated ventral prostate growth (figure 12). When an oral dose of 50 mg *E. ulmoides *extract was co-administered together with 5000 μg of IM testosterone (n = 5 for each combination treatment), the mean ventral prostate weight increased to 93 mg/100 g body weight, demonstrating a highly significant δ difference over baseline of 53 mg/100 g body weight (p-value < 0.001). The outcome of these animal studies further indicates that *E. ulmoides *potentiates the effect of testosterone on the androgen receptor (figure 12).

**Figure 13:**
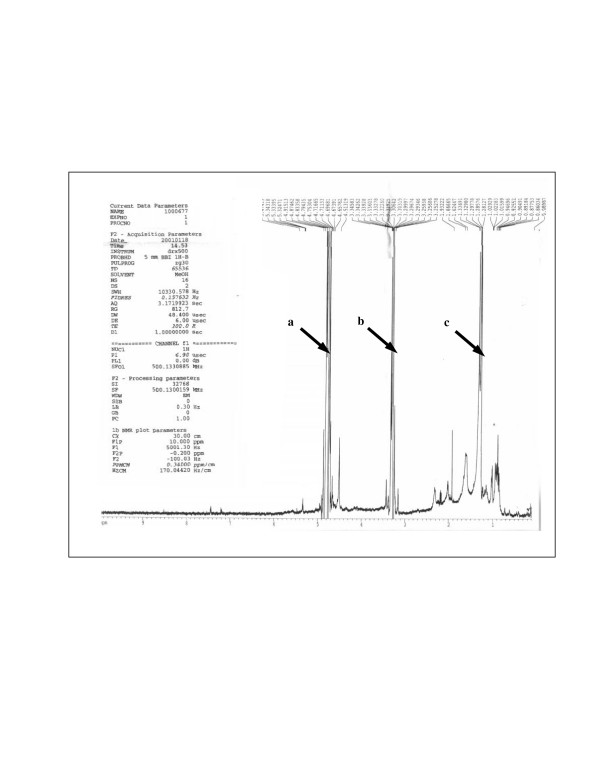
The ^1^H NMR spectra of supra-hormonal fraction CB. Arrow a shows protons from water (^1^H chemical shift of 4.8 – 5 ppm). Arrow b shows protons from the solvent methanol (^1^H chemical shift of 3.2 – 3.5 ppm). Arrow c shows protons from fatty acids (^1^H chemical shift of 0.8 – 1.8) ppm.

**Figure 14:**
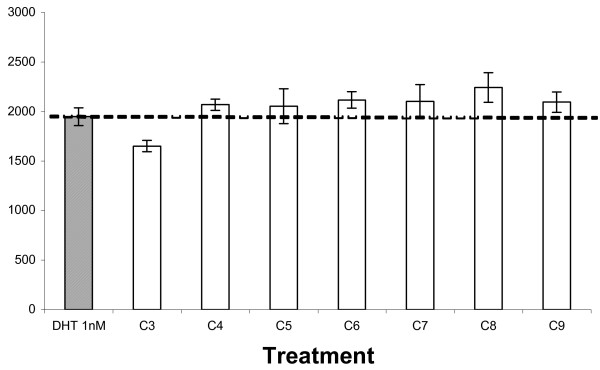
Effect of short-chain polysaturated fatty acids (PFAs) homologous series (C3 – C9) on androgen-dependent AR transactivational capacity. The effect of short chain (C3 – C8) polysaturated fatty acids (PFAs) on androgen dependent AR transactivational capacity. Hela cells tranfected with AR expression plasmid were exposed to 10 μM of different PFAs as indicated, together with 1 nM DHT. C3 = propanoic acid (3:0). C4 = butanoic acid (4:0). C5 = pentanoic acid (5:0). C6 = n-caproic acid (6:0). C7 = heptanoic acid (7:0). C8 = caprylic acid (8:0). C9 = pelargonic acid (9:0). AR activity was measured with the multimeric AR reporter plasmid (ARE_2_-TATA-Luc) and luciferase activity in relative light units (RLU) was mean ± SE of 3 replicates.

Subsequent ^1^H NMR and GC analyses of active fraction CB showed the major presence of the 8-carbon polysaturated fatty acid, caprylic acid, along with other lipids (figure 13 and table 1). Bioassays using pure caprylic acid and other polysaturated fatty acids (PFAs) correlated with the augmenting effect of *E. ulmoides *on the AR (figure 14) in varying degrees. Ethanolic extract of coconut (*Cocos nucifera*) flesh, rich in C-8 caprylic acid and other polysaturated fatty acids [11], replicated the hormone potentiating effect of both *E. ulmoides *extract and pure caprylic acid in AR bioassays (data not shown).

**Table 1:** Composition of major fatty acids in Fraction CB by standard gas chromatography (GC) analysis.

**Fatty acid**	**Carbon number and type of bond**	**Abundance (%)**
Caprylic acid	8:0	78.1977
Palmitic acid	16:0	9.0703
Stearic acid	18:0	6.4948
Palmitalic acid	16:1ω 9	1.5445
Linoleic acid	18:2ω 6	0.7221
Linolenic acid	18:3ω 3	0.6503
Subtotal		96.6797
Minor fatty acids	-	3.3203

**Total**		**100**

Caprylic acid, palmitic acid and stearic acid are polysaturated fatty acids (PFA). Palmitalic acid, Linoleic acid and Linolenic acid are omega-9, omega-6 and omega-3 polyunsaturated fatty acids (PUFA) respectively. These fatty acids share the general empirical formula C_n_H_n_O_n_. Caprylic acid was the major constituent with 78.2% abundance.

Okadaic acid, a known phosphorylation promoter, is able to strongly augment androgen-dependent AR activity [12]. Interestingly, fatty acids can also promote phosphorylation. One instance is oleic acid, a C-18 cis-monosaturated fatty acid [13]. It is possible that AR and ER augmentation by both *E. ulmoides *extract and caprylic acid arise from a common tripartite synergism between the steroid receptors, sex steroids and fats, based on a phosphorylation mechanism.

Our studies suggest intrinsic hormone potentiating effect in PFAs *per se*; in addition to the calorific and structural roles that have previously been identified (for e.g. cell membrane components or structural precursors of pro-inflammatory cytokines). The tripartite synergism demonstrated in this study may also be helpful in clarifying the known epidemiological link between high dietary saturated fat intake (rich in PFAs), obesity and increased risks of hormone-related diseases such as prostate or breast cancer [14].

## Conclusion

The novel discoveries reported in this study add phytoandrogens and lipidic augmenters to the emerging list of hormomimetics (such as phytoestrogens) known to exist in plants. Pharmaceutical utility of lipidic augmenters in the treatment of hypogonadal conditions such as menopause or andropause could be exploited based on this mechanism of tripartite synergism. The link between excess dietary lipids, hyperandrogenism and hormone-related disorders should also be further explored in the light of these findings.

## Competing interests

The author(s) declare that they have no competing interests.

## Authors' contributions

OYC: Designed and performed the study along with drafting the manuscript.

BTKH: Supervised and guided aspects of the study along with the drafting of the manuscript.

Both authors have read and approved the contents of this paper.

## Pre-publication history

The pre-publication history for this paper can be accessed here:


